# Sleep and Delirium in Pediatric Critical Illness: What Is the Relationship?

**DOI:** 10.3390/medsci6040090

**Published:** 2018-10-10

**Authors:** Amy Calandriello, Joanna C. Tylka, Pallavi P. Patwari

**Affiliations:** 1 Pediatric Critical Care Medicine, Rush Children’s Hospital, Rush University Medical Center, 1750 W. Harrison Street, Chicago, IL 606012, USA; Amy_E_Calandriello@rush.edu (A.C.); Joanna_C_Tylka@rush.edu (J.C.T.); 2 Pediatric Sleep Medicine, Rush Children’s Hospital, Rush University Medical Center, 1750 W. Harrison Street, Chicago, IL 606012, USA

**Keywords:** Acute illness, children, circadian disturbance, mechanical ventilation, melatonin, non-pharmacologic management, pediatric intensive care unit, screening, sedation

## Abstract

With growing recognition of pediatric delirium in pediatric critical illness there has also been increased investigation into improving recognition and determining potential risk factors. Disturbed sleep has been assumed to be one of the key risk factors leading to delirium and is commonplace in the pediatric critical care setting as the nature of intensive care requires frequent and invasive monitoring and interventions. However, this relationship between sleep and delirium in pediatric critical illness has not been definitively established and may, instead, reflect significant overlap in risk factors and consequences of underlying neurologic dysfunction. We aim to review the existing tools for evaluation of sleep and delirium in the pediatric critical care setting and review findings from recent investigations with application of these measures in the pediatric intensive care unit.

## 1. Introduction

Both disturbed sleep and delirium are notoriously difficult to recognize in the pediatric population and recognition becomes more challenging in the pediatric intensive care unit (PICU) when the underlying disease process and the administered medications contribute to alterations in level of consciousness. Further, during the acute phase of illness, primary goals for maintaining patient stability and safety focus on level of sedation rather than promoting sleep. There have been recent improvements in recognizing delirium in the hospitalized pediatric patient, particularly with validated screening tools, and increased attention to promoting sleep in the PICU setting. However, delirium screening and sleep promotion by pediatric intensivists are not widely applied internationally [1]. Despite advances to promote natural/physiologic sleep to prevent or treat delirium, the cause-effect relationship of sleep and delirium has yet to be clearly established. It may be that both dysregulated sleep and delirium are “sister” disorders that indicate underlying neurologic dysfunction.

## 2. Defining Delirium

The key feature of delirium is an alteration in both cognition and arousal that can have hypoactive or hyperactive subtypes. The American Psychiatric Association’s Diagnostic and Statistical Manual of Mental Disorders, Fifth Edition (DSM-5) defines delirium as a noticeable change in the patient’s neurocognitive baseline with an acute disturbance in attention, awareness, and cognition, and is thought to be a direct result of another medical condition rather than due to an established/evolving neurocognitive disorder [2] Additionally, clinical presentations of delirium can vary among pediatric patients and present in three different subtypes: Hyperactive, hypoactive, and mixed. Hyperactive delirium is characterized as agitation and aggression [3]. Hypoactive delirium is identified as a decrease in mental status and lethargy [3]. Mixed delirium, commonly referred to as emerging delirium, will manifest with both clinical signs of hyperactive and hypoactive delirium [3]. Pediatric patients in the critical care setting are predisposed to metabolic and environmental risk factors for delirium such as infection, withdrawal, disturbed sleep, immobility, noise disturbances, and sensory overload [4]. Delirium is a severe complication of pediatric critical illness associated with negative patient outcomes such as mortality, morbidity, and increased medical costs up to fourfold as a result of increased length of hospitalization [4,5].

## 3. Introduction of Validated Pediatric Delirium Screening Tools

Early detection of delirium can decrease long-term consequences related to neurocognitive impairment, inattentiveness, post-traumatic stress disorder, and spatial or verbal memory disturbances [3]. This has led to a recognition of the importance of screening for, diagnosing, and treating delirium including the creation of guidelines. The American College of Critical Care Medicine published guidelines for adult patients recommend routine monitoring of delirium in intensive care unit (ICU) patients [6]. Additionally, The European Society of Paediatric and Neonatal Intensive Care (ESPNIC) recommends that delirium be assessed and documented every 8–12 h [7]. However, as of the time of this publication, there are no guidelines for diagnosing delirium in pediatric intensive care units in the Unites States.

There are many challenges to detecting and diagnosing delirium in the PICU. First, diagnosis requires knowledge and a high index of suspicion by providers [8]. Second, the fluctuating nature of delirium can make it very difficult for providers that only spend short periods of time with the patient. Next, it can be difficult to differentiate between delirium, iatrogenic withdrawal syndrome, pain, and under-sedation as many of the symptoms overlap. Finally, the vast differences in neurocognitive development in infants and children make detecting delirium in young and developmentally delayed patients particularly challenging [9]. Recognition of delirium can be increased through use of a screening tool. An ideal screening tool for delirium would detect all three subtypes of delirium in patients of all ages and all developmental levels; accounting for the wide range of developmental milestones that occur and the severity of illness. Additionally, it would need to be quick, reliable, sensitive and specific. Multiple screening tools have been developed and validated to assist in identifying delirium in the pediatric intensive care population each with advantages and drawbacks as seen in Table 1.

## 4. Delirium Screening Tools

The Delirium Rating Scale (DRS) is one of the first screening tools for delirium to be developed [10]. It was designed to be used by psychiatrists and is labor intensive. However, in addition to detecting delirium, it can be used to determine delirium severity and follow severity over time. Though designed for adult patients, a retrospective study in PICU patients found the scale to be applicable [11]. The first tool specifically designed for the pediatric population, the Pediatric Anesthesia Emergence Delirium (PAED) scale was designed to detect emergence delirium following anesthesia [12]. While not developed for the PICU population, it has been applied in this population with notably poor sensitivity [13]. This is likely due to the fact that the questions focus on symptoms of the hyperactive subtype and thus may under detect the mixed and hypoactive subtypes. The Cornell Assessment of Pediatric Delirium (CAP-D) was developed from the PAED [14]. It is the tool recommended by the ESPNIC as it is simple, quick, and requires minimal training prior to implementation [7]. Another advantage of the CAP-D tool is that it has identified subtle clinical signs of hypoactive delirium which has been associated with worse clinical outcomes in pediatric critical care [15]. However, while sensitivity was retained in the developmentally delayed population the specificity decreased significantly.

The Pediatric Confusion Assessment Method (pCAM-ICU) was adapted from the most widely used adult delirium screen for children 5 years and older [16]. It can be completed by any provider as it does not require a prolonged interaction time with the patient, making it ideal for screening patients frequently or when suspicions arise. However, it requires patients to have the cognitive development of a 5-year-old and be cooperate with the screening, which limits utility. The pCAM-ICU also requires more extensive training of the provider than other screening tools. The Severity Scale for the Pediatric Confusion Assessment Method for the ICU (sspCAM-ICU) took the pCAM-ICU and added a scoring system [13]. This increases the sensitivity of the original screen, but also makes it more difficult to implement. Finally, The Preschool Confusion Assessment Method for the ICU (psCAM-ICU) is an adaptation of the pCAM-ICU for patients six months to five years of age [17]. It has many of the same advantages and disadvantages of the pCAM-ICU, however it does allow for assessment of some patients with developmental delays. 

Recognizing that withdrawal symptoms overlap with delirium, Ista et al. developed the Sophia Observation withdrawal Symptoms-Paediatric Delirium scale (SOS-PD) [18]. The SOS-PD is designed to be completed by the bedside nurse after a minimum of 4 h of interaction with the patient and is quick to complete and easy to implement. However, its greatest advantage is its ability to detect both delirium and withdrawal due to overlap of in symptoms such as anxiety, agitation, irritability, and disturbed sleep.

At this time no screening tool has emerged as superior. Validation studies have all been single center, small to medium sized with differences in designs that make it difficult to compare the screening tools. More research is warranted on screening tools. In particular, research that compares these tools across multiple centers and accounts for baseline developmental stage, withdrawal symptoms, and sleep disturbances [9]. Further, the existing pediatric delirium tools have limited evaluation of sleep disturbance and include a single perspective such as sleep-wake cycle [11,17], sleep duration [18], or restlessness [12,14].

## 5. Delirium in Pediatric Intensive Care Unit Patients

Within the last few years, there has been a robust increase in publications focused on pediatric delirium, as seen in Table 2. Studies included in this review were found through searching the electronic databases, PubMed and Scopus, using the key words: pediatric, delirium, and critical care. Criteria for inclusion was primary research focused on delirium with a primary cohort in the PICU or pediatric cardiac intensive care unit (CICU). Finally, further articles were found based on references from the primary searches. These studies have yielded a large amount of information on associations of patient characteristics, treatment modalities, and outcomes with delirium in the critically ill pediatric population.

Many studies have assessed associations between patient characteristics such as age, gender, severity of illness, reason for admission, and developmental delay in order to identify risk factors for development of delirium with varying results. Nine studies looked for associations between delirium and age with eight studies finding an association [5,15,19,20,21,22,23,24,25]. However, two found that delirium was associated with older age (>12 years) [19,20], while the other six found that delirium was associated with associated with younger age (<2, <5, or 2–5 years) [5,15,21,22,23,24]. Interestingly, the two studies that found associations with older age used exams by a psychiatrist to diagnose delirium while the other studies used screening tools (CAP-D or psCAM-ICU). These differences in the methods of diagnosing delirium may contribute to the inconsistent results and underscores the need for a consistent tool to screen and diagnose delirium in the PICU. 

Gender has been investigated in multiple studies; four studies found no association [5,20,21,22], with one study noting an association between the male gender and delirium [25]. Results have also been inconsistent with respect to severity of illness and delirium; three studies noted an association [23,24,26], but two found no association [25,27]. Pertaining to reason for admission, two studies found no association between reason for admission and delirium [5,20], while a study focusing on post-operative patients found a much higher incidence of delirium (66%) indicating that the post-operative status may be a risk factor for developing delirium [22]. Finally, developmental delay has been associated with delirium. Both Traube et al. [24] and Silver et al. [21] found on multivariate analysis that those with developmental delay had over 3 times the odds of developing delirium as those with normal development (odds ratio (OR) 3.31 and 3.45 respectively). 

Many treatment modalities also have been found to have associations with delirium including extracorporeal membrane oxygenation (ECMO), red blood cell (RBC) transfusions, anticholinergic medications, antiepileptic medications, benzodiazepine administration, and mechanical ventilation. Other treatment modalities have either been found to not have an association or have an unclear association with delirium including opioid medications, vasopressor medications, cyanotic heart disease, and cardiopulmonary bypass time. A study by Patel et al. [28] of pediatric patients requiring ECMO found that all eight patients developed delirium during their course. While this study is small, the 100% incidence must certainly add ECMO to the list of risk factors for delirium. Another treatment that has been associated with risk for delirium is RBC transfusions. Nellis et al. [29] found that children who received RBC transfusions had more than twice the incidence of delirium as children who were never transfused. Medications that have been associated with delirium in the PICU setting on multivariate analysis include anticholinergics [24], antiepileptics [5], and benzodiazepines.

Benzodiazepines are routinely used in pediatric critical illness with the intention to alleviate anxiety and ensure safety with invasive interventions. Recent studies have found higher frequency of delirium with benzodiazepine exposure in this population [5,23,24,30]. Traube et al. used the CAP-D tool and found that delirium was significantly more likely with benzodiazepine exposure from both a point prevalence study and a prospective longitudinal study [5,24]. Using the CAP-D tool, Mody et al. also found benzodiazepine exposure (but not opiates) to be an independent risk factor for development of delirium with more than a fourfold increase in transitioning from a normal mental status to a delirious state [30]. This association has been more closely evaluated in a narrower pediatric age group (0.5–5 years old) using the psCAM-ICU with findings of a non-linear increase in delirium frequency the day after benzodiazepine exposure and greater duration of delirium [23].

Respiratory failure requiring intubation and mechanical ventilator support is another risk factor for delirium independent of benzodiazepine exposure [5,15,20,21,24,25,30]. Traube et al. found that of 29% of 642 patients that ever required mechanical ventilation developed delirium as compared to 9% of 905 patients with delirium who never received mechanical ventilation [24]. Interestingly, in a pediatric CICU, the authors found no statistically significant differences in frequency of delirium based on type of respiratory support even comparing those who were ever mechanically ventilated (64% with delirium) to never mechanically ventilated (42% with delirium) during the admission, but found a statistically significant association between delirium and length of mechanical ventilator support [15]. This cohort of 99 patients in a pediatric CICU had a high incidence of delirium (57%) compared to general PICU cohorts and with a subgroup analysis found delirium to be more likely in children with cyanotic heart disease (26 of 36 patients with delirium) as compared to non-hypoxic cardiac defects (26 of 52 patients) [15].

Opioid medications on the other hand have mostly been found to not be a risk factor for delirium, which is based on multivariate analysis from three different studies [23,24,30]. Traube et al. [5], however, did find that patients with opioid exposure had twice the odds of delirium as those children without opioid exposure. Vasopressor medications have been found to be both associated with [5] and not associated with delirium [24]. While apparently contradictory results, it may be that vasopressor medications are used as a marker for disease severity rather than independently being a risk factor. Finally, both cardiopulmonary bypass time [15] and cyanotic heart disease [23] have been found not to be risk factors for delirium in single studies, contradicting the findings of Alverez et al. [15].

Delirium in the PICU population has been associated with several negative outcomes. A large number of studies have found an association with delirium and increased length of stay [5,15,20,22,23,24,25]. Additionally, delirium has been associated with increased duration of mechanical ventilation in two studies [15,25]. Smeets et al. [20] found an increase in direct medical costs of 1.5% due to pediatric delirium. Similarly, Traube et al. [31] noted that the median total PICU costs were significantly higher in patients with delirium than in patients who were never delirious. Further, after controlling for confounding factors (age, gender, severity of illness, and PICU length of stay), delirium was associated with an 85% increase in PICU costs. Finally, Traube et al. [24] found a significant increase in in-hospital mortality for children with delirium (5.24% vs. 0.94%). This persisted even after controlling for probability of mortality at admission (using the Pediatric Index of Mortality-3 score); the odds of mortality for those ever delirious was 4 times that of the never delirious group (OR = 4.39).

Less is known about the longer-term outcomes of the pediatric critical care population however a few studies have been published with encouraging results. Schieveld et al. [19] noted that delirium resolved in all study patients and that 38 of the 40 patients (95%) were successfully treated with antipsychotic medications. Similarly, Barnes et al. [32] found that 81% of patients diagnosed with delirium were prescribed antipsychotic medications, with only 23% (3 of 13) being discharged on these medications. Meyburg et al. [33] found no association between delirium in the PICU and long-term cognition or behavior based on follow-up questionnaires and exams preformed 12 to 24 months after the delirium event.

## 6. Sleep in Pediatric Intensive Care Patients

Patients in the intensive care unit are at increased risk for significant disturbance in sleep quality and quantity and for alteration of the sleep-wake pattern, which is generally accepted as unavoidable. Exogenous influences upon sleep in the PICU include environmental factors (light, noise, intrusive monitoring and intervention) and medications. Endogenous influences can be attributed to the underlying disease process such as hypoxia, respiratory failure, sepsis/inflammation, central nervous system injury including traumatic brain injury, and pain. Short-term sleep deprivation is known to affect behavior and chronic long-term sleep disturbances affect neurocognitive development in children—both important factors to consider for improving short and long term outcomes during recovery from critical illness. For this review, “sleep disturbance” is a non-specific term that refers to changes from baseline of total sleep time, sleep architecture, and circadian rhythm. In other words, sleep disturbance includes inadequate total sleep time, sleep fragmentation (frequent arousals), variance in the quantity and distribution of sleep stages (particularly slow wave sleep and rapid eye movement (REM) sleep), and circadian rhythm disturbance (or circadian misalignment). Underlying or pre-existing primary sleep disorders such as obstructive sleep apnea or hypoventilation (sleep related breathing disorders), narcolepsy (hypersomnolence disorders), and sleep related movement disorders are not included in the scope of this review. 

The most challenging issue is defining and measuring sleep disturbance in critical illness, which is complicated by variable neurocognitive baseline, distinguishing sedated state from normal sleep stages, constant physiologic changes over a 24 h period, and determining the correct timing for evaluation. Polysomnography (PSG), the gold-standard for measuring sleep, is not a reasonable tool for prolonged monitoring; Alternative, objective measures of sleep are limited in accuracy and usefulness in the PICU. For example, actigraphy is not reliable in patients who are heavily sedated or paralyzed, serial melatonin levels would be impractical and difficult to interpret, limited montage electroencephalogram (EEG) is also cumbersome and time-intensive, and bispectral index monitoring cannot distinguish normal sleep from sedated state.

The number of studies that have evaluated sleep in pediatric critical illness have variable methodology, variable aims, and with only a few studies that utilized limited montage EEG assessment of sleep [34], which limits broad application of findings. Further, bedside evaluation of sleep is not reliable. Armour et al. closely compared observer assessment and PSG data in 40 pediatric burn patients and found that patient sleep is often falsely over-estimated with 56.3% false-positive rate and 96.5% true-positive rate [35]. In day-to-day practice, evaluation of sleep in the PICU setting is based on bedside nursing assessment since the gold-standard for objective evaluation of sleep with polysomnography (PSG) is cumbersome, expensive, and of limited utility with non-24 hour recording.

Two studies in the pediatric critical care setting found fragmented sleep and absence of diurnal variation [36,37]. Carno evaluated sleep via PSG recording in 2 mechanically ventilated children under neuromuscular blockade and found that sleep was fragmented, demonstrated variance in sleep stage distribution as compared to published normal values, and that a large proportion of sleep occurred during the day. Further, sleep was variable from day-to-night and from day-to-day indicating significant circadian disruption. Marseglia et al. [38] also found altered circadian rhythm in mechanically ventilated children based on repeated measures of serum melatonin. 

Only one study directly evaluated sleep with polysomnogram and medication intervention. The authors evaluated sleep and hormone response to zolpidem or haloperidol in pediatric burn victims and found that both medications improve sleep continuity; zolpidem increased stage N3 (slow wave) sleep and REM sleep and haloperidol increased total sleep time and stage N2 sleep [39].

## 7. Sleep and Delirium Relationship in the Intensive Care Unit

A direct relationship of sleep disturbance and delirium has not been evaluated in pediatric critical illness. What we can glean from studies in adult patients is that the evidence that sleep disturbance leads to delirium is not clearly established. With the specific aim of determining if improving sleep is associated with reduction in delirium, Flannery et al. [40] performed a systematic review and found 6 of 10 studies reported statistically significant reduction in adult ICU delirium with sleep promotion, but only 3 of these 6 studies included a sleep assessment. Of the 10 studies, sleep assessment was included in only 4 studies which were based on patient report, which would not be feasible in a patient with delirium who would be dependent on bedside observations of sleep [40]. Patel et al. [41] were the only authors who found a decrease in delirium along with improvement in sleep measures in adult patients in the ICU setting; the intervention group was given non-pharmacologic treatment with reduction in light, noise, and disturbance during the night and found clinically and statistically significant reduction in delirium incidence from 33% (55 of 167 patients) prior to intervention to 14% (24 of 171 patients) after intervention. A recent Cochrane review regarding sleep promotion in adult ICU settings looked specifically at non-pharmacologic interventions and found low quality evidence for an effect on objective and subjective sleep measures (including patient satisfaction), delirium risk, length of ICU stay, and adverse events; Though, meta-analysis from two studies demonstrated a lower incidence of delirium and improved total sleep time based on nurse observation with use of earplugs and eye masks [42].

**Figure 1:**
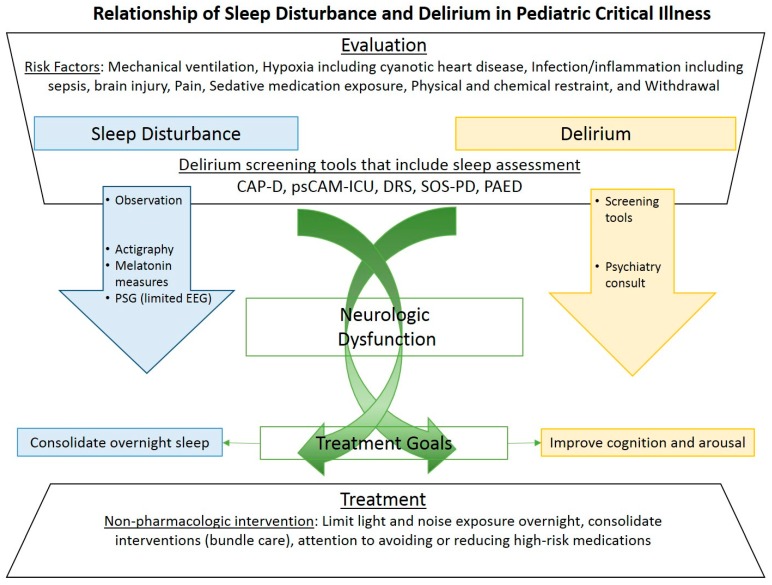
The first step in evaluating sleep disturbance and delirium are recognizing the similar risk factors. Next, identification of sleep disturbance and delirium are distinct except for a few delirium screening tools. Distinct measurements for sleep disturbance and delirium are noted by the large arrows with associated treatment goals noted by the corresponding color boxes. Finally, noted at the bottom, non-pharmacologic intervention for both sleep disturbance and delirium are the same. Abbreviations: polysomnogram (PSG) with limited electroencephalogram (EEG).

Both sleep disturbance and delirium have similar risks and management in the ICU setting, as seen in Figure 1. Risks includes hypoxia, mechanical ventilation, infection/inflammation, central nervous system (CNS) injury, pain, exposure to sedative medications, and withdrawal. Regarding sleep disturbance, frequent interventions, intubation with mechanical ventilation, and pain can contribute to inadequate sleep time, sleep fragmentation, and disrupt the normal progression of sleep states. Sedative medications will affect the quantity and distribution of sleep stages such that slow wave sleep and REM sleep are significantly reduced (opioids are known to reduce slow wave sleep; benzodiazepines are known to suppress REM sleep). Further, systemic infection and inflammation such as sepsis can lead to circadian rhythm disturbance. Mentioned previously, risk factors for delirium have significant overlap to these risk factors for sleep disturbance. Further, due to the complex nature of critical illness, dissecting out the specific cause of acute change in neurocognition and awareness is extremely difficult. 

Both delirium and sleep involve a change in mental state—one is pathologic and the latter is physiologic. Evaluation of changes in mental state are challenging in critically ill children. Extrapolating from adult studies and applying to pediatric patients has limitations that include increase use of sedation (younger children cannot safely tolerate intubation or other invasive intervention without significant anxiety and high risk for self-harm), inability for accurate self-report of sleep quantity and quality, and significant changes in sleep characteristics in the first decade of life (duration, pattern, sleep stage distribution). Even with these limitations, non-pharmacologic strategies to improve sleep are reasonable based on low risk and possible benefit. While not easily measured in the PICU, the underlying justification for sleep promotion is that sleep is inherently restorative (particularly slow wave and REM sleep), improving “sleep” can potentially reduce the amount of sedative required, and can improve pain tolerance [43].

## 8. Treatment

Strategies to prevent both sleep disruption and delirium should be the first step in management. This includes non-pharmacologic interventions to reduce noise and light exposure overnight and bundled nursing interventions to limit sleep fragmentation. Other strategies would include attention to medications that affect sleep (through changes in sleep stage distribution or circadian rhythm) and pose higher risk for development of delirium; this includes benzodiazepines, opioids, steroids, beta-blockers, dopamine, and norepinephrine. Lastly, routinely used medications for sleep promotion in pediatric patients is limited to melatonin, while pharmacologic agents for delirium include antipsychotic agents. 

There have been many studies in the adult ICU population on non-pharmacologic strategies for the prevention of delirium. Prominent among them is a multicomponent program known as the Hospital Elder Life Program (HELP) which has shown great success in reducing delirium in the elderly population [44]. This program prioritizes regular cognitive orientation, therapeutic activities, sleep enhancement, early mobilization, vision and hearing adaptations, fluid repletion, and feeding assistance. A recent meta-analysis of this program found that 14 studies demonstrated significant reductions in delirium incidence (OR 0.47) [44]. Unfortunately, while these strategies hold great promise, they have yet to be shown to be effective outside of the elderly population. The adult guidelines recommend early mobilization of patients as a delirium prevention strategy, but make no further recommendations on either pharmacological or non-pharmacological methods [6]. In the PICU population, Simone et al. [25] implemented a multidisciplinary bundle that included establishing daily routines, encouraging parental involvement, orientation, creating a familiar environment, reducing restraint use, and creating an uninterrupted sleep environment between 11 p.m. and 4 a.m. This bundle’s implementation was associated with a reduction in delirium from 19.3% to 11.8% [25]; encouraging results that underscore the need for further studies. 

The primary role of intrinsic melatonin is to regulate the circadian rhythm (sleep-wake timing), but other potential effects including, but not limited to, neuroprotective and anti-inflammatory roles are particularly relevant to critical illness. Investigation of exogenous melatonin use in pediatric critical illness has not been completed, but non-standardized administration of melatonin is common during the recovery period in PICU to facilitate sleep onset. A recent Cochrane review evaluated the effect of melatonin on sleep in adult ICU patients and included 4 studies that met sufficient criteria for inclusion. Of these, the authors found no significant effect on subjective and objective report of sleep quality and quantity, no significant difference in anxiety, mortality, or length of stay.

Pharmacologic agents used to treat pediatric delirium include typical and atypical antipsychotics. Haloperidol has been used successfully in the PICU on agitated and delirious patients [45], however, given its side effect profile it has been largely superseded by other options with lower risks. Atypical antipsychotics that have been used in the treatment of pediatric delirium include olanzapine, risperidone, and quetiapine. Turkel at al. [46] found a comparable response between these three medications in a retrospective review of 110 patients with delirium. Additionally, mean DRS (revised-98) scores were significantly decreased with treatment [46]. Further, quetiapine was found to be safe for short term use in this population in a retrospective review of 50 patients [47]. A final area that should be considered when treating PICU patients with delirium is choice of sedation medications. Benzodiazepines in particular should be reduced or discontinued. Dexmedetomidine is recommended over benzodiazepines for sedation infusions in the adult guidelines based on results of two randomized controlled trials [6]. While studies in the pediatric population are lacking, this approach may be helpful in treating pediatric delirium. 

## 9. Conclusions

Both delirium and sleep disturbance are problems in the PICU population that have been increasingly recognized in recent years. Delirium has been associated with a number of adverse outcomes including increased length of stay [5,15,20,22,23,24,25], increased duration of mechanical ventilation [15,25], increased medical costs [20,31] and increased in-hospital mortality [24]. Non-pharmacologic interventions for promoting sleep and reducing delirium in the PICU setting has low risk, low cost, and potential benefit and should be the initial focus for all children at risk for delirium. Overall, there is no evidence of a direct cause-effect relationship between sleep disturbances and delirium in children, and it may be that improvement in sleep occurs in parallel with reduction in delirium. Therefore, attention to both improving sleep and preventing delirium are equally important in pediatric critical illness. 

Regarding management strategies, we recommend the following actions to prevent, diagnose and treat delirium in the PICU. The first step in reducing and treating delirium is early recognition, thus screening all PICU patients with a validated delirium screening tool should be completed every 8–12 h. We recommend implementing environmental strategies to promote sleep and decreased delirium that include establishing a daily routine with attention to sleep and wake time, reducing light and noise exposure during sleep times (by encouraging reduced room lighting and eliminating electronic media exposure), encouraging parental involvement and attention to having familiar items in the environment, reducing arousals and awakenings with bundled-care during sleep time, frequent age-appropriate re-orientation for the child, reducing restraint use, and early mobilization. Medications should be reviewed to reduce those associated with delirium (if appropriate and feasible), particularly benzodiazepine use. While, it is not yet clear if melatonin administration leads to meaningful and/or positive outcomes, it should be considered when circadian misalignment (sleep wake disturbance) is suspected. We recommend the use of melatonin in patients with positive delirium screen and suspected sleep disturbance as it is a relatively benign medication with potential benefit. After early recognition of delirium based on screening tools, collaboration and support from pediatric psychiatry should be encouraged for guiding pharmacotherapy in the critically ill child; particularly confirmation of diagnosis and support in deciding if antipsychotic medications would be helpful as pediatric intensivists have less experience with use of antipsychotic medications. 

For future directions, given that research on sleep and delirium in the PICU population is far behind what has been done in the adult population, more investigation is needed. The first step is establishing a standardized, accurate, and simplified tool to detect both sleep disturbance and delirium. Significant progress has been made with the emergence of validated delirium screening tools for use in pediatric patients; however, multicenter studies and comparison studies are needed to establish which screening tool best serves the pediatric population with critical illness and account for the broad developmental spectrum. Objective, cost-effective measures for sleep has been the greatest limiting step for progress with understanding causes and consequences of sleep disturbance (quality, quantity, and pattern) in the ICU setting, and thus requires future attention to accurately characterize sleep as well as delineate the relationship between sleep and delirium. Further, it would be ideal to determine which interventions have the greatest impact on improving both sleep and reducing delirium. Use of melatonin supplementation is theoretically promising but use in pediatric critical illness still needs to be clarified regarding who, when, and how long administration would be needed. Finally, it is imperative to have future investigations with focus on treatments, both pharmacologic and non-pharmacologic, as well as short and long term outcomes. To our knowledge, this is the first review to address the parallel between sleep disturbance and delirium in pediatric patients affected by critical illness and to emphasize the entwined roles rather than a cause-effect relationship.

## Figures and Tables

**Table 1:** Advantages and Limitations of Pediatric Delirium Screening Tools.

Tool	How It Works	Validation Study *	Population	Sensitivity **	Specificity **	Interrater Reliability (κ) ***	Observation Time for Score	Sleep Assessment	Pros	Cons
Cornell Assessment of Pediatric Delirium (CAP-D) [14]	8 questions rated on a scale of 0–4 based on interactions with patient over shift	111 patients Prospective	0–21 years + Intubated patients + Develop-mentally delayed RASS score −3 or greater	94%	79%	0.94	Once per shift	1 question assesses restlessness	Takes less than 2 minutes to complete	Decreased specificity in develop-mentally delayed children
Pediatric Confusion Assessment Method (pCAM-ICU) [16]	4 step screen with 2 steps requiring patient interaction squeezing hand, nodding or answering yes/no	68 patients Prospective	5 years and older + Intubated patients RASS score −3 or greater	83%	99%	0.96	None specified	None	Screen identifies if patients have required (DSM) delirium features	Must have cognitive development of 5 years of greater May require tools (cards/pictures)
Severity scale for the Pediatric Confusion Assessment Method for the ICU (sspCAM-ICU) [13]	Adds a point system to the pCAM-ICU	64 patients Prospective	5 years and older + Intubated patients	85%	98%	Not assessed	None specified	None	Performed better than pCAM-ICU in direct testing	Complex scoring system
Preschool Confusion Assessment Method for the ICU (psCAM-ICU) [17]	4 step screen with 1 step requiring patient to look at picture/cards	300 patients Prospective	6 months–5 years + Intubated patients	91%	75%	0.79	None specified	1 step assesses sleep-wake cycle	Screen identifies if patients have required DSM delirium features	Requires tools (cards/ pictures) Not able to be used on develop-mentally delayed children of children with visual/audi-tory impairments
Delirium Rating Scale (DRS) [11]	10 items scored on a scale from 0 to 4	84 patients Retrospective #	6 months–19 years	N/A	N/A	N/A	24 h	1 of the 10 items assess sleep-wake cycle	Scale has been validated in adults [10]	Unable to assess sensitivity, specificity or interrater reliability due to retrospective design
Sophia Observation withdrawal Symptoms- Paediatric Delirium scale (SOS-PD) [18]	22 item yes or no check list based on at least 4 h with patient	146 patients	3 months to 16 years +Intubated patients COMFORT scale ≥ 11	97%	92%	0.9	Once per shift (minimum of 4 h with patient)	1 question assesses the length of sleep	Also detects withdrawal	Only patients with positive SOS-PD screens were seen by psychiatrist (may miss patients)
Pediatric Anesthesia Emergence Delirium (PAED) scale [12]	5 items rated on a scale of 1–4	64 patients [13] Prospective	5 years and older +Intubated patients	69%	98%	0.8	None specified for pediatric intensive care unit (PICU) setting	1 question assesses restlessness	Simplest screening tool with just 5 questions	Designed for post-anesthesia emergence delirium

* All validation studies were single center studies in PICUs; ** Compared with diagnosis of delirium by a psychiatrist using Diagnostic and Statistical Manual of Mental Disorders (DSM; version DSM-III-R or DSM-IV depending on time); *** Cohen’s κ coefficient; # Retrospective study of patients diagnosed with delirium; PICU: pediatric intensive care unit.

**Table 2:** Delirium in Pediatric Intensive Care Unit Patients.

Authors (year)	Study Design	Age (yrs)	Number of Patients	Delirium Frequency	Measurement/Tool Used	Risk Factors Associated with Delirium *	Risk Factors NOT Associated with Delirium *	Outcomes
Schieveld et al. (2007) [19] and Schieveld et al. (2008) [26] **	Single center, Prospective, descriptive study	0–18	877 61 with possible delirium	5% of total population 66% of suspected population (*n* = 40)	Exam by a pediatric neuropsychiatrist using Diagnostic and Statistical Manual of Mental Disorders, Fourth Edition, (DSM-IV) criteria	Older Age (>12 years) Severity of illness (Pediatric Index of Mortality and Pediatric Risk of Mortality scores)	N/A	All patients fully recovered from delirium
Smeets et al. (2010) [20]	Single center, Prospective, descriptive study	1–18	49 diagnosed with delirium 98 randomly selected patients without delirium	N/A	Exam by a neuropsychiatrist using DSM-IV criteria.	Older Age Mechanical Ventilation	Gender Reason for admission	Delirium was associated with increased length of hospitalization and medical costs.
Silver et al. (2015) [21]	Single center, Prospective observational study	0–21	99	21%	Cornell Assessment of Pediatric Delirium (CAP-D) twice daily	Developmental delay Mechanical ventilation Preschool age (2–5 years)	Severity of illness (Pediatric Index of Mortality 2) Gender	N/A
Traube et al. (2016) [31]	Single center, Prospective observational study	0-21	464	16% (*n* = 74)	CAP-D twice daily	N/A	N/A	After controlling confounding factors, delirium was associated with an 85% increase in PICU costs
Meyburg et al. (2017) [22]	Single center, prospective, observational study	0–17	93 post elective surgical patients	66% (*n* = 61)	CAP-D twice daily	Postoperative state Younger age	Gender	Delirium was found to be a predictor of increased hospital length of stay.
Patel et al. (2017) [28]	Single center, Prospective observational longitudinal cohort study	0.6–16	8 patients requiring ECMO	100% (*n* = 8)	CAP-D daily	Extracorporeal membrane oxygenation (ECMO)	N/A	Only 13% of days on ECMO were delirium and coma free Delirium screening was successfully completed on 97% of ECMO days
Simone et al. (2017) [25]	Single center, Prospective observational study	0–21	1875	17% (*n* = 140)	CAP-D	Male Gender Mechanical Ventilation	Severity of illness (Pediatric Index of Mortality 2) Age	PICU length of stay, hospital length of stay, and duration of mechanical ventilation were significantly longer in patients with delirium than without
Smith et al. (2017) [23]	Single center, Prospective observational study	0.5–5	300	41% (*n* = 124)	psCAM-ICU	Benzodiazepine exposure Younger age (<2 years) Severity of illness (Pediatric Risk of Mortality Score at admission)	Cyanotic Heart disease Opioid exposure Mechanical Ventilation Lowest Oxygen saturation	Delirium increased length of hospitalization in pre-school aged patients.
Traube et al. (2017) [24]	Single center, Prospective, longitudinal cohort study	0–21	1547	17% (*n* = 267)	CAP-D twice daily	Younger age (<2 years) Developmental delay (Pediatric Cerebral Performance Category 4) Severity of illness (Pediatric Index of Mortality-3 score) Mechanical ventilation Benzodiazepine exposure Anticholinergic exposure	Opioid exposure Corticosteroid exposure Vasopressor medication exposure	PICU length of stay was increased in children with delirium Delirium was a strong and independent predictor of mortality
Traube et al. (2017) [5]	Multi institutional, international point-prevalence study	0–21	835 (develop-mentally delayed children excluded)	25%	CAP-D	Younger age (<2 years) Mechanical ventilation Benzodiazepine exposure Opioid exposure Antiepileptic exposure Vasopressor medication exposure	Reason for admission Gender Ethnicity	Delirium was associated with a prolonged length of stay (>5 days)
Alvarez et al. (2018) [15]	Single center, Prospective observational cohort study	0–21	99 total patients screened after admission to cardiac intensive care unit (CICU) >12 h	57% (*n* = 56)	CAP-D twice daily	Younger age Mechanical ventilation Benzodiazepine exposure	Cardiopulmonary bypass time	Delirium is associated with increased length of mechanical ventilation and increased length of hospital stay.
Barnes et al. (2018) [32]	Single center, Retrospective chart review	2–20	50 patients who received child psychiatry consult	32% (*n* = 16)	Exam by a pediatric psychiatrist using DSM-IV criteria	N/A	N/A	81% (*n* = 13) of the patients with delirium were prescribed an antipsychotic.
Meyburg et al. (2018) [33]	Single center point prevalence study	1–16	47 patients diagnosed with delirium	N/A	CAP-D twice daily	N/A	N/A	No association between pediatric delirium and long-term cognition or behavior.
Mody et al. (2018) [30]	Single center, Retrospective observational study	0–18	580	23% (*n* = 131)	CAP-D daily	Benzodiazepine exposure Mechanical ventilation	Opioid exposure	The strongest predictor of delirium was being delirious on the day prior
Nellis et al. (2018) [29]	Single center, Nested retrospective cohort study within prospective cohort study	0–21	1547 total 166 patients who received RBC transfusion	17% (*n* = 267)	CAP-D twice daily	Transfusion of RBCs	Anemia (nadir hemoglobin) Age of blood transfused	N/A

* Associations based on multivariate analysis if completed; ** Both studies were completed on the same group of patients.
